# Intrinsic Brain Functional Activity Abnormalities in Episodic Tension-Type Headache

**DOI:** 10.1155/2023/6560298

**Published:** 2023-05-24

**Authors:** Xiu Yang, DianXuan Guo, Wei Huang, Bing Chen

**Affiliations:** ^1^Department of Neurology, The Affiliated Huaian No. 1 People's Hospital of Nanjing Medical University, Huaian, China; ^2^Department of Geriatrics, The Affiliated Huaian Hospital of Xuzhou Medical University, Huaian, China; ^3^Department of Medical Imaging, The Affiliated Huaian No. 1 People's Hospital of Nanjing Medical University, Huaian, China

## Abstract

**Objective:**

The neurobiological basis of episodic tension-type headache (ETTH) remains largely unclear. The aim of the present study was to explore intrinsic brain functional activity alterations in ETTH.

**Methods:**

Resting-state functional magnetic resonance imaging (rs-fMRI) data were collected from 32 patients with ETTH and 32 age- and gender-matched healthy controls (HCs). Differences in intrinsic brain functional activity between patients with ETTH and HCs were analyzed utilizing the fractional amplitude of low-frequency fluctuation (fALFF) approach. Correlation analyses were performed to examine the relationship between fALFF alterations and clinical characteristics.

**Results:**

Compared to HCs, patients with ETTH exhibited increased fALFF in the right posterior insula and anterior insula and decreased fALFF in the posterior cingulate cortex. Moreover, the fALFF in the right anterior insula was negatively correlated with attack frequency in ETTH.

**Conclusions:**

This study highlights alterations in the intrinsic brain functional activity in the insula and posterior cingulate cortex in ETTH that can help us understand its neurobiological underpinnings.

## 1. Introduction

Tension-type headache (TTH) is the most common but least studied form of primary headache disorders. It is estimated that about 1.89 billion individuals are affected globally that cause major disability and significant socioeconomic impact [1]. TTH is usually characterized by a dull, tight, or squeezing band-like pain sensation around the head. Despite its high prevalence, its pathophysiology remains incompletely understood. Increasing evidence suggests that abnormalities in the central nervous system may play a role in the pathophysiology of TTH. A magnetoencephalographic study found altered excitability of the primary somatosensory cortex in TTH [2]. Structural magnetic resonance imaging (MRI) studies have revealed that TTH is associated with gray matter (GM) volume abnormalities [3–5] and white matter (WM) alterations [6] in widespread brain regions.

In the last two decades, resting-state functional magnetic resonance imaging (rs-fMRI) based on the blood-oxygen-level-dependent (BOLD) signal has been widely utilized as a promising tool to quantitatively characterize regional brain function, functional connectivity, and functional networks both in healthy subjects and patients with neuropsychiatric disorders [7]. The amplitude of low-frequency fluctuation (ALFF) is a metric for rs-fMRI data that is thought to reflect spontaneous brain activity [7, 8]. ALFF measures the regional intensity of spontaneous fluctuations in the BOLD signal within a typical frequency range (e.g., 0.01–0.1 Hz) [7, 8]. A decreased ALFF in TTH was observed in the middle frontal gyrus and superior gyrus [9]. Specifically, fractional ALFF (fALFF) is an alternative technique for ALFF standardization. Compared with ALFF, fALFF detected signals more sensitively and specifically as it has been shown to be less susceptible to nonspecific physiological noise [10]. Many studies have demonstrated abnormal spontaneous brain activity indexed by fALFF in migraine [11] and many other chronic pain disorders [12–14]. Although peripheral pain mechanisms probably play an important role in episodic TTH (ETTH), increasing evidence has shown the involvement of central mechanisms in its pathophysiology [3, 15]. However, to the best of our knowledge, no study using fALFF has been performed to elucidate the neural mechanisms underlying ETTH.

In the present study, we conducted the first whole-brain voxel-wise fALFF analysis of rs-fMRI data to assess intrinsic brain activity abnormalities in patients with ETTH relative to healthy controls. We also conducted exploratory analyses of the relationships between the brain fALFF abnormalities and clinical variables of ETTH.

## 2. Materials and Methods

### 2.1. Participants

The current study recruited 32 adult patients with ETTH and 32 age- and gender-matched healthy controls (HCs). Of note, the sample included in this study partially overlapped with that in our previous work [5]. The patients with ETTH were diagnosed based on the third edition of the International Classification of Headache Disorders (ICHD-3) (codes 2.1 and 2.2) [16]. Only those who had not undergone treatment for at least three months and were diagnosed with ETTH were eligible for enrollment [5]. Exclusion criteria were as follows: (1) under age 18 or over age 65; (2) prior history of other types of headaches or other chronic pain disorders in the previous year; (3) a history of psychiatric disorders, addiction disorders, or major systemic or other neurologic diseases; (4) current use of any analgesics or any other regular medication; and (5) abnormal signal in the brain in conventional MRI scanning. Age- and gender-matched healthy controls (HCs) that did not suffer from ETTH and met the same exclusion criteria as the ETTH patients were recruited from the local communities. The study has received research ethics board approval, and written informed consents were obtained from all participants before the study.

Demographic and clinical information, including age, gender, education level, illness duration, headache intensity (visual analogue scale [VAS]), attack frequency (number/month), Zung Self-Rating Anxiety Scale (SAS) [17], and the Zung Self-Rating Depression Scale (SDS) [18] were collected from all eligible patients. Details of the demographic and clinical characteristics of both groups are presented in Table 1.

### 2.2. MRI Data Acquisition

Whole brain rs-fMRI data were acquired on a 3.0T scanner (Siemens Verio, Erlangen, Germany) with a standard head coil while subjects stayed awake and kept their eyes closed and their mind blank. The rs-fMRI data were acquired with an echoplanar imaging (EPI) sequence as follows: repetition time (TR)/echo time (TE) = 2000/30 ms, flip angle = 90°, in-plane matrix = 64 × 64, field of view = 240 × 240 mm, slice thickness = 3 mm, interslice space = 0 mm, NEX = 1, voxel size = 3.8 mm × 3.8 mm × 3 mm, and slice number = 36. Axial scans were parallel to the anterior-posterior commissure (AC-PC) line. rs-fMRI data were acquired from patients with ETTH during their pain-free periods.

### 2.3. Data Preprocessing and fALFF Calculation

Preprocessing of rs-fMRI data was performed with the toolbox for data processing and analysis for brain imaging (DPABI) [19] based on statistical parametric mapping (SPM12, http://www.fil.ion.ucl.ac.uk/spm/) running in MATLAB (version 2018b, MathWorks Inc., MA, United States). The procedures included the following steps: (1) conversion of image data format from DICOM to NIFTI; (2) removal of the first 10 scan volumes; (3) slice timing; (4) head motion correction (exclusion criteria: displacement >2.0 mm or angular rotation >2.0° in any direction); (5) nuisance covariates regression using the Friston 24-parameter model and additional regression of white matter, cerebrospinal fluid, and global brain mean signals; (6) coregistration and spatial normalization to Montreal Neurological Institute (MNI) space; (7) temporally band pass filtering (0.01–0.1 Hz); and (8) spatial smoothing with a 4-mm full-width at half-maximum Gaussian kernel. Microhead movements were controlled at the group level by taking the mean framewise displacement (FD) derived from Jenkinson's formula as a covariate [20].

After rs-fMRI data preprocessing, fALFF was calculated using the DPABI software [19]. The filtered time course of each voxel was first converted into the frequency domain using fast Fourier transform analysis. The power spectrum was then measured. The square root of the power of each voxel within the low-frequency range (0.01–0.1 Hz) is further computed as ALFF. The fALFF value of the image was calculated as the ratio of the low-frequency power spectrum to the power spectrum of the entire frequency range. For standardization, the subject-level voxel-wise fALFF was divided by the global mean fALFF value within the default mask [10].

### 2.4. Statistical Analysis

Two-sample *t*-tests were used to compare age, education level, FD, SAS, and SDS between the ETTH group and the HC group. A chi-square test was applied to compare gender differences between the two groups. fALFF differences between groups were compared using a two-sample *t*-test with age, gender, education level, SAS, SDS, and FD as covariates. According to the recent recommendation for multiple comparisons, the significance was determined using a permutation test with threshold-free cluster enhancement (TFCE) family-wise error (FWE) corrected *p* < 0.05.

To explore the relationship between abnormal neural activities and clinical variables (VAS, illness duration, and attack frequency), Spearman's correlation analyses were performed. The threshold of significance was set at *p* < 0.05 with Bonferroni's correction.

## 3. Results

### 3.1. Demographic and Clinical Characteristics of the Study Population

There were no statistical differences in age (*p* = 0.15), gender (*p* = 0.15), education level (*p* = 0.15), or FD (*p* = 0.15) between patients with ETTH and HCs. Increased scores in SAS and SDS were found in patients with ETTH compared with HCs (*p* < 0.05) (Table 1).

### 3.2. Group Differences in fALFF

**Figure 1:**
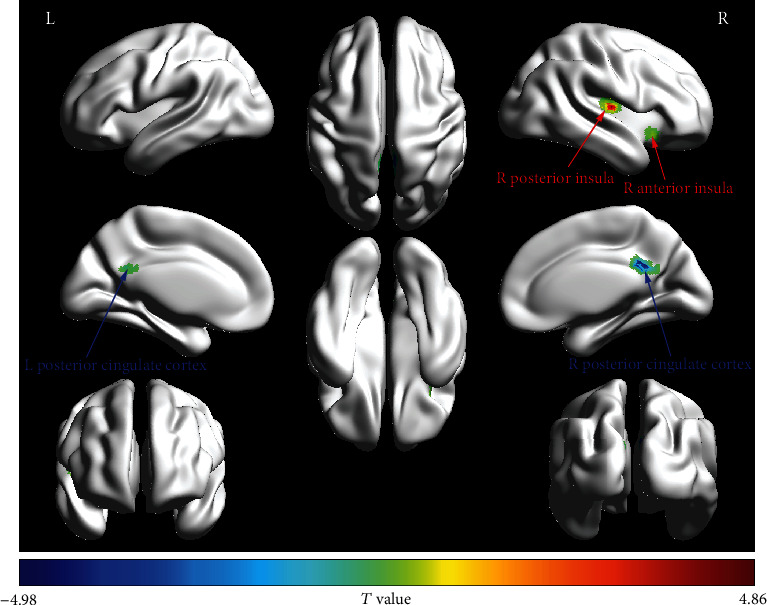
fALFF map for the group-level comparison (ETTH vs. HC). fALFF: fractional amplitude of low-frequency fluctuation; ETTH: episodic tension-type headache; HC: healthy control; L: left; R: right.

Compared with the HC group, the ETTH group showed significantly increased fALFF in the right posterior insula and anterior insula and decreased fALFF in the posterior cingulate cortex (PCC) after controlling for the effects of age, gender, education level, SAS, SDS, and FD (*p* < 0.05, TFCE-FWE corrected). The details of the fALFF differences are displayed in Figure 1 and Table 2.

### 3.3. Correlation Analysis

**Figure 2:**
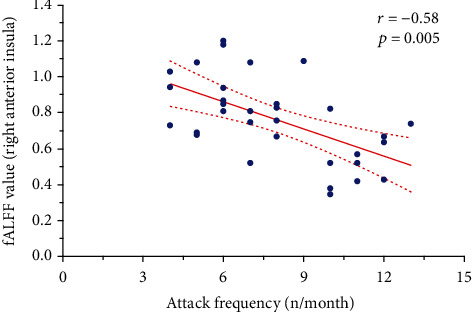
Relationship between the attack frequency and the fALFF values of the right anterior insula in the ETTH group. fALFF: fractional amplitude of low-frequency fluctuation; ETTH: episodic tension-type headache.

The fALFF values of the right anterior insula were negatively correlated with attack frequency (*r* = −0.58, *p* = 0.005; Figure 2). No significant correlations were found between altered fALFF and other clinical variables, including VAS and illness duration.

## 4. Discussion

To the best of our knowledge, this is the first study to use the fALFF approach by analyzing rs-fMRI data to explore intrinsic brain functional activity in ETTH. The present study demonstrates increased fALFF in the right posterior insula and anterior insula and decreased fALFF in the PCC in patients with ETTH relative to HCs. Moreover, the fALFF in the right anterior insula was negatively correlated with attack frequency. Our findings indicate altered spontaneous brain activity as a key feature underlying the central mechanisms of ETTH.

In the present study, we observed significantly increased fALFF of the right posterior insula and anterior insula in patients with ETTH compared with HCs. The insula is an anatomical integration hub that has widespread connections with brain cortical and subcortical regions [21–23]. In particular, the posterior insula participates in the sensory discriminative aspects of pain, while the anterior insula preferably processes the cognitive-affective components of pain [21–24]. Serving as the primary interoceptive cortex, the insula integrates multimodal salient information ranging from sensation to cognitive-affective events to create conscious interoception [24–26]. Disrupted interoceptive processing is fundamental to the perception, modulation, and chronification of pain [24, 27]. Given its prominent role, the insula is considered a potential target for therapeutic neuromodulation for chronic pain [28, 29]. Our current study using rs-fMRI data provided further evidence of the insula in the involvement of pain processing in ETTH during the pain-free phase. Although no structural abnormalities were observed in the pain-free phase, cortical plasticity in the anterior insula and other pain-related brain regions between the pain and pain-free phases were identified in patients with ETTH [5]. These findings suggest that the functional changes may have preceded structural abnormalities of the brain in ETTH. A prior study using voxel-based morphometry analysis demonstrated a significant gray matter decrease in the anterior and posterior insula as well as other brain regions in chronic TTH [4]. Notably, our study showed a negative correlation between the fALFF of the right anterior insula but not that of the posterior insula and attack frequency in ETTH. Several lines of evidence support the critical role of the anterior insula in the process of pain chronification [30, 31]. We thus speculated that the reorganization of intrinsic brain activity in the right anterior insula observed in our study may be one of the potential compensatory mechanisms in the dynamic evolution of TTH chronification, although it is warranted to be further investigated.

Another notable finding of our study was that patients with ETTH showed decreased intrinsic neural activity in the PCC relative to HCs. This is in alignment with studies that identified decreased PCC function in acute and chronic pain states [13, 32]. Structural MRI also showed evidence of gray matter reduction in the PCC in chronic TTH [4]. Meta-analyses of functional imaging studies have demonstrated the association between pain and the rostral part of the PCC [33]. The PCC is the central core of the default mode network (DMN) that is involved in higher-level functions such as attention, memory, prospection, and self-processing and is posited to be implicated in internally cognitive-attentional dimensions of pain [13, 34]. Numerous studies have reported significant functional changes within the DMN and functional connectivity alterations between the DMN and other brain regions/networks (particularly the insula/salience network) in a variety of chronic pain conditions [13, 35–37], which was considered a marker for pain itself [13]. These convergent lines of evidence implicate PCC in the neurobiological underpinnings of TTH.

Our and three other prior studies to date have investigated spontaneous neural activity in TTH; however, they showed a lack of consensus on the findings [9, 15, 38]. The inconsistencies among these studies may be possibly due to the differences in the analytical methods (ReHo/ALFF/fALFF), sample heterogeneity (episodic TTH/mixed samples with episodic and chronic TTH), medication, and/or sample size. The fALFF metric has also been utilized to investigate spontaneous neural activity in other types of headaches, including migraine and cluster headache. In migraineurs, changes in fALFF were observed in the bilateral middle frontal gyrus, left postcentral, and right lingual gyrus [39], as well as in the left rostral ventromedial medulla, right thalamus, left amygdala, and right angular gyrus [40]. Patients with migraine without aura exhibited alterations in fALFF in the inferior parietal gyrus, bilateral precentral gyrus, right postcentral gyrus, bilateral supplementary motor areas, bilateral precuneus, and left superior frontal gyrus/medial prefrontal cortex [11], as well as in the ventral posteromedial thalamus and brainstem [41]. Those with vestibular migraine demonstrated fALFF changes in the right postcentral gyrus, right superior parietal gyrus, right supplementary motor area, and superior frontal gyrus [42]. In contrast, patients with left cluster headache displayed fALFF alterations in the left cerebellum, left lentiform nucleus, left frontal lobe, left anterior cingulate, and right postcentral gyrus, while those with right cluster headache exhibited fALFF changes in the right cerebellum, right cingulate gyrus, right superior parietal lobule, right inferior parietal lobule, right postcentral gyrus, and left precuneus [43]. These findings have contributed to a better understanding of the neural mechanisms underlying these disorders. Our results are novel, expand previous findings related to aberrant spontaneous neural activity in TTH, and further support the central role in the pathophysiology regardless of episodic or chronic TTH.

Although our findings are encouraging, some limitations need to be acknowledged. The sample size of the patients included in our study was relatively small. Our results are preliminary and require validation on a larger dataset. In addition, our study only focused on the fALFF alterations during the pain-free phase in patients with ETTH. Further longitudinal studies may be helpful to evaluate dynamic functional changes and to elucidate the neural plasticity in different phases of this disorder. In addition to spontaneous neural activity, brain functional networks and multimodal MRI data should be further examined that may provide more insights into the neural mechanism of the disease.

## 5. Conclusions

Our study revealed resting-state intrinsic neuronal reorganization in the insula and PCC in treatment-naïve patients with ETTH in their pain-free phase, which provides a new perspective for exploring brain abnormalities in ETTH and contributes to further understanding its underlying central mechanisms.

## Figures and Tables

**Table 1:** Demographic information and clinical characteristics of the study population.

	ETTH patients	HC	*P* value
Gender (male/female)	14/18	15/17	0.8
Age (years)	34.03 ± 9.34	33.44 ± 7.29	
Education (years)	11.47 ± 2.87	11.16 ± 2.85	0.66
Illness duration (years)	4.88 ± 3.02	—	—
Attack frequency (times/months)	7.94 ± 2.66	—	—
VAS (0–100)	41.88 ± 11.02	—	—
SAS	43.81 ± 5.01	29 ± 6.13	<0.001
SDS	33.59 ± 5.03	30.06 ± 5.85	0.012
FD	0.22 ± 0.13	0.2 ± 0.067	0.5

TTH: episodic tension-type headache; HC: health controls; VAS: visual analogue scale; SAS: Zung Self-Rating Anxiety Scale; SDS: Zung Self-Rating Depression Scale; FD: framewise displacement. Data is presented as mean and standard deviation.

**Table 2:** Summary of fALFF differences between patients with ETTH and healthy controls.

Brain regions	Brodmann areas	Maximum MNI coordinates (*x*, *y*, *z*)	Cluster size	*T* value^∗^
Right posterior insula	BA 48	42, -14, 11	165	4.86
Right anterior insula	BA 38	38, 18, -12	35	3.61
Posterior cingulate cortex	BA 23	0, -48, 28	180	-4.98

fALFF: fractional amplitude of low-frequency fluctuation; ETTH: episodic tension-type headache; BA: Brodmann area; MNI: Montreal Neurological Institute. ^∗^The significance was determined using a permutation test with threshold-free cluster enhancement family-wise error corrected *p* < 0.05.

## Data Availability

All the data that support the conclusions of this article are available from the corresponding author upon reasonable request.
